# Noble Metal Nanoparticles Applications in Cancer

**DOI:** 10.1155/2012/751075

**Published:** 2011-10-05

**Authors:** João Conde, Gonçalo Doria, Pedro Baptista

**Affiliations:** ^1^CIGMH, Departamento de Ciências da Vida, Faculdade de Ciências e Tecnologia, Universidade Nova de Lisboa, Campus de Caparica, 2829-516 Caparica, Portugal; ^2^Instituto de Nanociencia de Aragón, Universidad de Zaragoza, Pedro Cerbuna 12, 50009 Zaragoza, Spain

## Abstract

Nanotechnology has prompted new and improved materials for biomedical applications with particular emphasis in therapy and diagnostics. Special interest has been directed at providing enhanced molecular therapeutics for cancer, where conventional approaches do not effectively differentiate between cancerous and normal cells; that is, they lack specificity. This normally causes systemic toxicity and severe and adverse side effects with concomitant loss of quality of life. Because of their small size, nanoparticles can readily interact with biomolecules both at surface and inside cells, yielding better signals and target specificity for diagnostics and therapeutics. This way, a variety of nanoparticles with the possibility of diversified modification with biomolecules have been investigated for biomedical applications including their use in highly sensitive imaging assays, thermal ablation, and radiotherapy enhancement as well as drug and gene delivery and silencing. Here, we review the available noble metal nanoparticles for cancer therapy, with particular focus on those already being translated into clinical settings.

## 1. Introduction

Cancer is one of the leading causes of mortality in the modern world, with more than 10 million new cases every year [1]. It is well established that cancer is a multifactorial disease caused by a complex mixture of genetic and environmental factors [2–4], where considerable advances have led to a more comprehensive understanding of cancer at the genetic, molecular, and cellular levels providing new targets and strategies for therapy [5]. Nevertheless, these advances have yet to be effectively translated into functioning diagnostics and therapy. For example, the effectiveness of many anticancer drugs is limited due to the inability to reach the target site in sufficient concentrations and efficiently exert the pharmacological effect without causing irreversible unwanted injury to healthy tissues and cells [6, 7].

The technological leap of controlling materials at nanoscale provides for a “big revolution” in medical and healthcare treatments and therapies [8, 9]. Nanotechnology offers a wealth of tools to diagnose and treat cancer—new imaging agents, multifunctional, targeted devices capable of bypassing biological barriers to deliver therapeutic agents directly to cells and tissues involved in cancer growth and metastasis, monitor predictive molecular changes allowing preventive action against precancerous cells, and minimizing costs and side effects [5, 10, 11]. Nanotechnology-based therapies for cancer with minimal side effects and high specificity are on the surge, where the main challenge is to develop a system for molecular therapy capable of circulating in the blood stream undetected by the immune system and recognize the desirable target, signaling it for effective drug delivery or gene silencing with minimum collateral cell damage—nanovectorization. As a result, personalized medicine could become a reality in cancer patient management.

**Figure 1:**
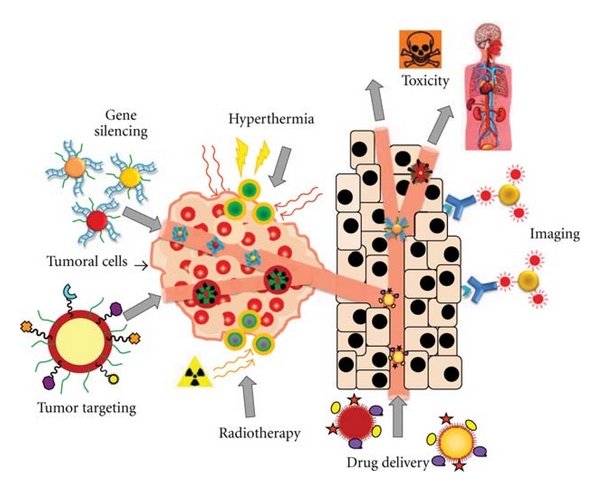
Noble metal NPs for cancer therapy. Once the tumor is directly connected to the main blood circulation system, NPs can exploit several characteristics of the newly formed vasculature and efficiently target tumors. Tumor cells are supplied by blood capillaries that perfuse the cells of the tissue where NPs can (i) passively accumulate or (ii) anchor through targeting moieties to biomarkers overexpress by tumor cells. NPs can act simultaneously as therapeutic agents, inducing hyperthermia, enhancing radiotherapy, silencing genes, and/or delivering drugs to induce tumor cell death, and as imaging enhancers or contrast agents, to help tracking the therapeutic effects in real time.

Nanoparticles (NPs), and noble metal NPs in particular, are versatile agents with a variety of biomedical applications including their use in highly sensitive diagnostic assays [12, 13], thermal ablation, and radiotherapy enhancement [14–17], as well as drug and gene delivery [18–21]. Moreover, noble metal NPs have been proposed as nontoxic carriers for dru and gene-delivery applications [22–24]. Additionally, the nanoparticle-based systems can provide simultaneous diagnostic and therapy, that is, Theranostics, exploring their unique properties for better penetration of therapeutic moieties and tracking within the body, allowing a more efficient therapy with a reduced risk in comparison to conventional therapies [25]—see Figure 1.

The unique characteristics of noble metal NPs, such as high surface-to-volume ratio, broad optical properties, ease of synthesis, and facile surface chemistry and functionalization hold pledge in the clinical field for cancer therapeutics [22, 26, 27]. Noble metal NPs (e.g., gold, silver, or a combination of both) present highly tunable optical properties, which can be easily tuned to desirable wavelengths according to their shape (e.g., nanoparticles, nanoshells, nanorods, etc.), size (e.g., 1 to 100 nm), and composition (e.g., core/shell or alloy noble metals), enabling their imaging and photothermal applications under native tissue [28, 29]. These NPs can also be easily functionalized with various moieties, such as antibodies, peptides, and/or DNA/RNA to specifically target different cells [30] and with biocompatible polymers (e.g., polyethylene glycol and PEG) to prolong their *in vivo* circulation for drug and gene delivery applications [23, 24]. Moreover, they can efficiently convert light or radiofrequencies into heat, thus enabling thermal ablation of targeted cancer cells [31, 32].

In this paper, we will focus on the application of noble metal NPs for cancer therapy with particular emphasis on their use *in vivo* and their potential to be translated into clinical settings.

## 2. Therapy

In medical terms, a therapeutic effect is a consequence of a medical treatment of any kind, the results of which are judged to be desirable and beneficial [33]. Conventional therapy methods in cancer involve the employment of agents that do not greatly differentiate between cancerous and normal cells, leading to systemic toxicity and adverse and severe side effects [34]. Efficient *in vivo* targeting to heterogeneous population of cancer cells and tissue still requires better selectivity and noncytotoxicity to surrounding healthy cells. However, universally targeting cells within a tumor is not always feasible, because some drugs cannot diffuse efficiently and the random nature of the approach makes it difficult to control the process and may induce multiple-drug resistance—a situation where chemotherapy treatments fail due to resistance of cancer cells towards one or more drugs [7]. Making use of their extraordinary properties, nanotechnology-based systems could offer a less-invasive alternative, enhancing the life expectancy and quality of life of the patient [35]. Among these, the potential therapeutic application of noble metal NPs represents an attractive platform for cancer therapy in a wide variety of targets and clinical settings [36, 37].

### 2.1. Tumor Targeting

It is expected that the greatest gains in therapeutic selectivity will be achieved by synergistic combinations of several multicomponent targeting strategies that is capable of simultaneously target and deliver multiple therapeutic agents while avoiding the organism's biological and biophysical barriers. NPs targeting strategies to cancerous tissues have focused on passive and active targeting. In passive targeting, because numerous tumors present defective vasculature and poor lymphatic drainage due to the rapid growth of solid tumors, noble metal NPs can extravasate into the tumor stroma through the fenestrations of the angiogenic vasculature, demonstrating targeting by enhanced permeation and retention, thus accumulation at the tumor site [6, 8, 38]. Additionally, functionalization of the NP's surface with hydrophilic molecules, such as PEG, can also greatly increase their solubility, help evading macrophage-mediated uptake and, thus, avoid removal from the systemic circulation and protect their carriers from enzymatic degradation when used *in vivo* [30]. For active targeting, NPs can be easily functionalized with a wide variety of biological moieties, such as antibodies, peptides, and/or DNA/RNA to specifically target extracellular and intracellular receptors or pathways [30]. The use of NPs functionalized with multiple peptides or antibodies, such as monoclonal antibodies, have been described to successfully target specific cell surface proteins or receptors on cancer cells and further direct their antitumor action, leading to tumor cell death with minimal damage to collateral healthy cells [36, 39–41]. In nucleic-acid functionalized NPs, DNA and RNA macromolecules can be used to simultaneously target specific sequences and exert their genetic-based therapy [42, 43].

To help tracking noble metal NPs *in vivo* and enhance the imaging properties of such moieties, leading to more efficient control of their therapeutic properties, they can also be functionalized with chemical moieties, such as Raman [44, 45] or fluorescent [46, 47] reporters.

### 2.2. Gene Silencing

Antisense DNA [48, 49] and RNA interference (RNAi) via the use of small-interfering RNA [50–53] have emerged as a powerful and useful tools to block gene function and for sequence-specific posttranscriptional gene silencing, playing an important role in downregulation of specific gene expression in cancer cells.

Small interfering RNAs (siRNAs) can be transfected into mammalian cells by a variety of methods that influence the strength and duration of the silencing response, which in turn is affected by the amount of siRNA that is delivered and on the potential of each siRNA to suppress its target. Thus, one drawback of using naked siRNAs is that they show extremely short half-lives, weak protection against action by RNases, poor chemical stability, and common dissociation from vector [54]. In fact, the major obstacle to clinical application is the uncertainty about how to deliver therapeutic RNAs (e.g., miRNA and/or siRNA) with maximal therapeutic impact. Nanotechnology offers an unprecedented opportunity to overcome these problems, as nanoscale devices, due to their small size, can readily interact with biomolecules on both the surface of cells and inside of cells for longer periods of time [10]. Gold NPs (AuNPs) have shown potential as intracellular delivery vehicles for antisense oligonucleotides [55] and for therapeutic siRNA by providing protection against RNAses and ease of functionalization for selective targeting [42, 43]. For example, Mirkin and coworkers showed that AuNPs attached to single-stranded oligodeoxynucleotides can be used for gene therapy, providing a highly efficient gene regulator in terms of high loading of the antisense DNA with no toxicity at the concentrations studied [55]. They have also shown that polyvalent RNA-AuNP conjugates are readily taken up by cells and that the particle bound siRNA could effectively regulate genes in the context of RNA interference [42]. AuNPs modified with the hydrophilic PEG polymer, siRNAs and then coated with poly(*β*-aminoester)s have been shown to facilitate high levels of *in vitro* siRNA delivery and gene silencing in human cells [56]. Also, Braun et al. developed an Au-nanoshell functionalized with TAT-lipid layer for transfection and selective release of siRNA [57], where the TAT-lipid coating was used to efficiently mediate the cellular uptake of the nanoconjugates and the siRNA release was dependent on near-infrared (NIR) laser pulses. The authors demonstrated that this NIR strategy for siRNA release was proficient and time dependent.

Several other studies using engineered NPs modified with siRNA have demonstrated a cytoplasmic delivery system of siRNA and efficient gene silencing using AuNPs [42, 56, 58–60].

### 2.3. Hyperthermia

Hyperthermia is based on the effect increasing temperatures have on living cells, and it is commonly accepted that above 42°C cell viability is strongly reduced. In fact, hyperthermia effects can range from moderate denaturation of blood and extracellular proteins to induction of apoptosis and, above 50°C, to cell death and tissue ablation [61]. Hyperthermia therapy in cancer has been widely used either via direct irradiation or suitable temperature vectors, such as metal NPs [62]. In nanoparticle-mediated hyperthermia for cancer, NPs heat up cancerous cells beyond their temperature tolerance limits, which are lower than normal healthy tissue due to their poor blood supply, killing them selectively. This can be achieved by exposing the entire patient or the targeted area to an alternating current magnetic field, an intense light source or radiofrequencies which will cause the NPs to heat up and induce thermal ablation of the tumor. One of the most widespread examples of hyperthermia mediated by NPs, magnetic NPs have been introduced in the body through magnetic delivery systems or local injection to the affected area [63]. The first *in vivo* Phase II clinical trials of magnetic NP hyperthermia were undertaken in Germany in 2005 [64] by injecting the prostate of cancer patients with biocompatible magnetite NPs. Successful results were obtained using minimally invasive ablation of the tumor in an AC magnetic field after several sessions.

Noble metal NPs have thoroughly been used as photothermal agents for *in vivo* therapy as a less invasive experimental technique that holds great promise for the treatment of cancer [65]. It combines two key components: (i) light source, such as lasers with a spectral range of 650–900 nm [66] for deep tissue penetration and (ii) optical absorbing NPs which efficiently transforms the optical irradiation into heat on a picosecond time scale, thus inducing photothermal ablation [67, 68]. For example, Huang and coworkers demonstrated that Au-nanorods are effective photothermal agents due to their longitudinal absorption band in the NIR on account of their SPR oscillations [65, 66, 69]. Small diameter Au-nanorods are being used as photothermal converters of near infrared radiation (NIR) for *in vivo* applications due to their high absorption cross-sections beyond the tissue absorption spectra. Since NIR light transmits readily through human skin and tissue, these nanorods can be used as ablation components for cancer [70, 71]. Other gold nanostructures such as Au-nanoshells [72–74], Au-nanocages [67, 75, 76], and spherical AuNPs [77] have also demonstrated effective photothermal destruction of cancer cells and tissue. PEG-modified Au-nanoshells (Silica/Au core/shell NPs) injected intravenously in tumor-bearing mice showed to passively accumulate in the tumor tissue due to the leakiness of the tumor vasculature. The rapid heating of Au-nanoshells upon NIR laser irradiation allowed for effective photothermal ablation of tumor in the mouse [78]. A similar approach was used by Terentyuk et al., where plasmonic silica/gold nanoshells were used to produce a controllable laser hyperthermia in tissues, thus enhancing the photothermal effect in cancer cells [79]. Sirotkina et al. described the use of AuNPs for skin tumor therapy based on local laser-inducing hyperthermia. After intravenous injection, the AuNPs accumulated in the skin tumor cells after 4-5 hours and induced apoptotic death of tumor cells, completely inhibiting the tumor growth after just five days of treatment [80]. 

The photothermal properties of AuNPs can also be used to generate transient vapor nanobubbles in order to produce a tunable nanoscale theranostic agent, described as plasmonic nanobubbles [81]. These nanobubbles are generated when the AuNPs are locally overheated with short laser pulses, due to the evaporation of a very thin volume of the surrounding medium, which in turn creates a vapor nanobubble that expands and collapses within nanoseconds. Plasmonic nanobubbles have been successfully applied as an *in vivo* tunable theranostic cellular agent in zebrafish hosting prostate cancer xenografts and in leukemia cells of human bone marrow specimens, presenting higher therapeutic selectivity when compared with AuNPs alone [82, 83]. The use of noninvasive radiowaves at 13.56 MHz have also been shown to induce heat in AuNPs and thermally destroy tumor tissue [84]. *In vivo* rat exposures to 35 Watts using direct AuNPs injections resulted in significant thermal injury at subcutaneous injection sites. Radio waves have the advantage of presenting significantly better penetration on tissue than NIR light, making them more efficient for deeper solid tumors [85]. Nonetheless, despite their greater depth of penetration, there is also greater energy attenuation by tissue.

Gold-silver-(AuAg-) nanorods labeled with molecular aptamers proved to require up to six orders of lower laser power irradiation to induce cell death when compared to Au-nanoshells or Au-nanorods [86]. These aptamer Scg8-AuAg-nanorods conjugates presented excellent hyperthermia efficiency and selectivity to CEM cells, exceeding the affinity of the original aptamer probes alone. Bimetallic AuAg-nanostructures with a dendrite morphology and hollow interior have also been developed as photothermal absorbers to destroy A549 lung cancer cells [87]. The photothermal performance of such dendrites required lower NP concentrations and laser power for efficient cancer cell damage when compared to Au-nanorods photothermal therapeutic agents. Likewise, Cheng and coworkers evaluated the photothermal efficiencies of three Au-based nanomaterials (silica@Au-nanoshells, hollow Au/Ag nanospheres and Au-nanorods) at killing three types of malignant cells (A549 lung cancer cells, HeLa cervix cancer cells, and TCC bladder cancer cells) using a CW NIR laser [88]. Silica@Au-nanoshells needed the lowest NP concentration for effective photo-ablation, whereas hollow Au/Ag nanospheres and Au-nanorods needed increasingly higher concentrations.

Gold has also been used together with magnetic or paramagnetic materials to enhance the photothermal effect and, thus, increase cancer cell death [89, 90].

### 2.4. Drug Delivery

The vast majority of clinically used drugs for cancer are low molecular-weight compounds that diffuse rapidly into healthy tissues being evenly distributed within the body, exhibit a short half-life in the blood stream and a high overall clearance rate. As a consequence, relatively small amounts of the drug reach the target site, and distribution into healthy tissues leads to severe side effects. Poor drug delivery and residence at the target site leads to significant complications, such as multidrug resistance [91]. As seen above, nanoparticles can be used as vectors for targeting cancer tissue/cells so as to optimize biodistribution of drugs. The NPs' performance as drug vectors depends on the size and surface functionalities of the particles, drug release rate, and particle disintegration. These systems show evidence of enhanced delivery of unstable drugs, more targeted distribution and capability to evade/bypass biological barriers.

AuNPs have already been used as vehicles for the delivery of anticancer drugs, such as paclitaxel- [92] or Platinum- (Pt-) based drugs (e.g., cisplatin, oxaliplatin, etc.) [93, 94]. Gibson et al. described the first example of 2 nm AuNPs covalently functionalized with the chemotherapeutic drug paclitaxel [92]. The administrations of hydrophobic drugs require molecular encapsulation, and it is found that nanosized particles are particularly efficient in evading the reticuloendothelial system [95]. Gold-gold sulfide nanoshells covered by a thermosensitive hydrogel matrix have been developed as a photothermal modulated drug-delivery system [96]. These nanoshell-composite hydrogels were designed to strongly absorb NIR light and release multiple bursts of any soluble material held within the hydrogel matrix in response to repeated NIR irradiation. More recently, Yavuz and coworkers developed a similar approach using 50-nm hollow Au-nanocubes (nanocages) with eight lopped-off porous corners covered by a thermosensitive polymer containing a preloaded effector that can be later released in a controllable fashion using an NIR laser [18].

### 2.5. Radiotherapy

Radiotherapy uses ionizing radiation for cancer treatment to control the proliferation of malignant cells. Nonetheless, the delivery of a lethal dose of radiation to a tumor while sparing nearby healthy tissues remains the greatest challenge in radiation therapy. Noble metal NPs can act as antennas, providing enhanced radiation targeting with lower radiation doses, consequently avoiding damage to healthy tissues. The irradiation may also be used to activate the NPs and set up the release of their cytotoxic action. AuNPs, upon X-ray irradiation, can act as dose enhancers and/or generate radicals that damage cancer cells and induce cell apoptosis and have been proposed as potential radiosensitizers for X-ray cancer therapy [97]. The use of this strategy has led to improvement in the treatment on cancer cells with little or no increase in harm to normal surrounding tissues in mice models [15] and also in breast cancer [98]. More recently, Xu and coworkers studied the potential effects on radiation-induced killing of glioma cells mediated by 10, 20, and 40 nm AuNPs and 20, 50, and 100 nm silver nanoparticles (AgNPs), all modified with proteins from fetal bovine serum [99]. Treating glioma cells with AgNPs led to radiation dose-dependent cytotoxicity, with smaller size particles (20 and 50 nm) being the most cytotoxic at relatively harmless radiation doses. In this study, AuNPs showed little effect on cell survival across different doses of ionizing radiation, which contrasted with the results of previous studies performed with AuNPs coated with PEG or amino acids in mice colorectal adenocarcinoma and breast cancer cells [15, 98]. Hypothetically, the different coatings of the AuNPs used may be responsible for the different outcomes observed. 

The use of platinum NPs (PtNPs) as prominent radiation sensitizers in radiotherapy cancer treatment showed strong enhancement of the biological efficiency of radiations, leading to amplified lethal damage in DNA from tumor cells, when compared to metal atoms [37].

## 3. Imaging

**Figure 2:**
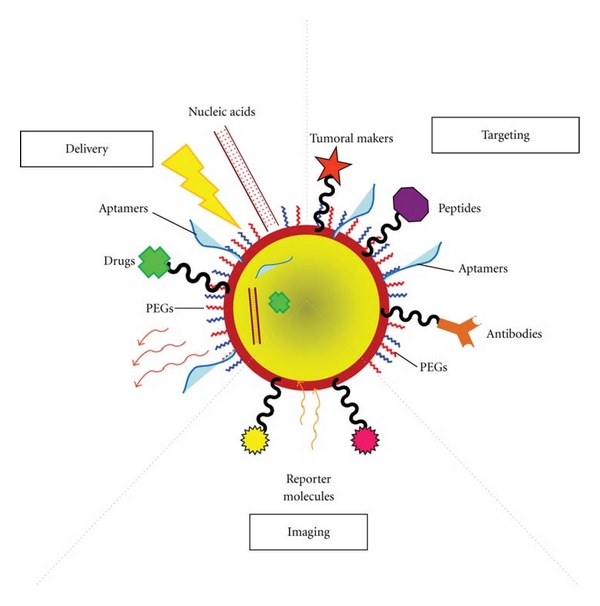
Multifunctional NP-based systems for tumor targeting, delivery and imaging. These innovative NPs comprise nucleic acids, aptamers and anticancer drug molecules for delivery to the target tissue. Depending on the targeting mechanism, they can be on the surface or inside the NPs. Responsive NPs/molecules can also trigger reaction upon external stimuli through the functionality of valuable tumor markers, peptides, polymers and antibodies that can used to improve NP circulation, effectiveness and selectivity. Multifunctional systems can carry reporter molecules tethered to the particle surface and employed as tracking and/or contrast agents.

Along with their therapeutic capabilities, most noble metal NPs can be used for the simultaneous actuation and tracking *in vivo*—see Figure 2. Because light absorption from biologic tissue components is minimized at near infrared (NIR) wavelengths, most noble metal NPs for *in vivo* imaging and therapy have been designed to strongly absorb in the NIR so as to be used as effective contrast agents [100]. However, noble metal nanomaterials, such as NPs, nanoshells, nanoclusters, nanocages, and nanorods, have showed widespread application as contrast agents for *in vivo* cancer imaging: those presenting a significant absorbance and scattering in the NIR region [46, 101] or surface-enhanced Raman scattering (SERS) [102], or as contrast agents for computed tomography (CT) [103], magnetic resonance imaging (MRI) [104], optical coherence tomography (OCT) [105–107], and photoacoustic imaging (PAI) [108]. Moreover, most noble metal nanomaterials are capable of combining multiple imaging modalities that can yield complementary information and offer synergistic advantages over any single imaging technique [109, 110].

Three-dimensional imaging can be achieved by computed tomography (CT), where a series of plane-cross-sectional images along an axis are interlinked by computer to create a 3D image. Typically, the cross-sectional images are acquired using X-ray radiation involving larger radiation doses than the conventional X-ray imaging procedures, which could lead to increased risk to public health [111]. The use of ~30 nm PEG-coated AuNPs for *in vivo* CT contrast agent was shown to increase image contrast, which allows to reduce the radiation dosage needed, allow to overcome the limitations of conventional contrast agents (e.g., iodine-based compounds), such as short imaging times due to rapid renal clearance, renal toxicity, and vascular permeation [103]. Hybrid NPs with a super-paramagnetic iron oxide/silica core and a gold nanoshell, with significant absorbance and scattering in the NIR region, have been used *in vivo* as dual contrast agents for CT and magnetic resonance imaging (MRI) presenting high CT attenuation and a good MR signal in hepatoma, compensating for the weakness of each modality [112].

Optical coherence tomography (OCT) is an imaging modality that provides cross-sectional subsurface imaging of biological tissue with micrometer scale resolution. The extra scattering achieved by using Au-nanoshells has been shown to provide an enhanced optical contrast and brightness for improved diagnostic imaging of tumors in mice due to the preferential accumulation of the nanoshells in the tumor. [78]. Tseng et al. developed nanorings with a localized surface plasmon resonance covering a spectral range of 1300 nm that produced both photothermal and image contrast enhancement effects in OCT when delivered into pig adipose samples [113]. Moreover, the image contrast enhancement effect could be isolated by continuously scanning the sample with a lower scan frequency, allowing to effectively control the therapeutic modality. Similarly, gold capped nanoroses have been used in photothermal OCT to detect macrophages in *ex vivo* rabbit arteries [114].

Photoacoustic imaging (PAI) and photoacoustic tomography (PAT) are noninvasive imaging techniques capable of resolving the optical absorption map of tissue at penetration depths akin with ultrasound imaging. Wang and coworkers have used this technique to image the distribution of Au-nanoshells circulating in the vasculature of a rat brain by achieving a gradual enhancement of the NIR optical absorption in the brain vessels [115]. These Au-nanocages enhanced the contrast between blood and the surrounding tissues by up to 81%, allowing a more detailed image of vascular structures at greater depths. Additionally, these nanocages were shown to be better suited for *in vivo* applications, specially due to their more compact size (<50 nm compared to >100 nm for Au-nanoshells) and larger optical absorption cross sections when compared to Au-nanoshells. Gold-nanorods show the maximum of the plasmon resonance tuned further into the NIR that allowed Motamedi et al. to develop a contrast agent for a laser optoacoustic imaging system for *in vivo* detection of gold nanorods and to enhance the diagnostic power of optoacoustic imaging [116]. Song et al. proposed a noninvasive *in vivo* spectroscopic photoacoustic sentinel lymph node mapping using gold nanorods as lymph node tracers in a rat model [117].

Also, noble metal NP probes can be used for *in situ* diagnostics of cancer. For example, NP-based NIR probes can overcome several limitations of conventional NIR organic dyes, such as poor hydrophilicity and photostability, low quantum yield and detection sensitivity, insufficient stability in biological systems, and weak multiplexing capability. Additionally, the high scattering properties of these NPs can enhance contrast of imaging systems based on microscopy, such as dark-field or dual-photon luminescence microscopy. Zhang et al. developed fluorescent metal nanoshells as molecular imaging agents to detect single microRNA (miRNA) molecules in lung cancer cells [47]. These metal nanoshells were composed of silica spheres with encapsulated Ru(bpy)_3_^2+^ complexes as core and thin silver layers as shell. The silver shell allowed to enhance emission intensity up to 6-fold and photostability by 2-fold, as well as to achieve longer lifetime emission signals that overcome cellular autofluorescence interference. Loo et al. demonstrated the use of NIR scattering Au-nanoshells as a contrast agent in dark-field microscopy to target antihuman epidermal growth factor receptor 2 (HER2), a clinically significant breast cancer molecular marker [72]. These Au-nanoshells were also used by Bickford et al. for imaging live HER2-overexpressing cancer cells using two-photon microscopy [118].

Surface-enhanced Raman scattering (SERS) using Au- or AgNPs with an attached reporter species with a Raman signature can be explored to highlight cellular structures and provide molecular structural information on the cellular environment in live cells [119, 120]. The use of such NPs allows for higher spectral specificity, multiplex capabilities, improved contrast and photostability to Raman-based imaging techniques. *In situ* monitoring of photothermal nanotherapy of LNCaP human prostate cancer cells by SERS was a significant enhancement of the Raman signal intensity by several orders of magnitude that have been observed [44].

## 4. Toxicity

Both *in vivo* and *in vitro*, nanoparticles have a tendency to accumulate within various types of cells with special affinity for macrophage-type cells (both histiocytes and blood phagocytic cells) and reticuloendothelial cells throughout the body. They also produce varying degrees of bioaccumulation in such tissues as lymph nodes, bone marrow, spleen, adrenals, liver, and kidneys [121–123]. The NPs size plays an important role in avoiding immune activation and renal clearance, thus enhancing their circulating time and availability for effective therapy. For example, hydrophilic NPs ranging in size between 10 and 100 nm are small enough to slow down activation of the mononuclear phagocyte system but are big enough to avoid renal filtration [8]. Research shows that NPs can stimulate and/or suppress the immune responses and that their compatibility with the immune system is largely determined by their surface chemistry. In fact, the influence of size, solubility, and surface modification on the biocompatibility of NPs and their use in biological applications is well known [122]. In terms of acute toxic effects to cells, noble metal NPs have been shown to induce DNA damage and oxidative damage [124–126].

Generally, AuNPs are considered to be benign, but the size similarity to biological molecules could provide “camouflage” to cellular barriers, leading to undesired cellular entry which might be detrimental to normal cellular function [127]. A systematic investigation of the size-dependent cytotoxicity of AuNPs against four cell lines found that 1 to 2 nm AuNPs displayed cell-type-dependent cytotoxicity with high micromolar IC50s, whereas 15 nm AuNPs were nontoxic to cells at concentrations 60-fold higher than the IC50 of the smaller AuNPs [128]. These results seemed to confirm size-dependent toxicity of AuNPs, an inference that has hitherto been shown to be somewhat ambivalent [129–134]. In fact, Yen et al. showed that AuNPs, especially those of smaller sizes, dramatically led to a decrease in the population of the macrophages and upregulated the expressions of proinflammatory genes interlukin-1, interlukin-6, and tumor necrosis factor alpha [135]. Sun et al. studied the *in vivo* toxicity of AuNPs according to their shape in KM mice showing that rod-shaped AuNPs were the most toxic, followed by cube-shaped AuNPs, while sphere-shaped AuNPs displayed the best biocompatibility, revealing that toxicity is shape dependent. Moreover, this study revealed that all AuNPs accumulated preferentially in the liver and spleen organs [136]. Nonetheless, it is worth pointing out that CTAB (a cationic surfactant commonly used for Au-nanorods synthesis) was also recently pointed out as the source of Au-nanorods' cytotoxicity, which may explain their toxicity in the previous studies [137].

Silver NPs are generally considered more toxic than AuNPs, with several studies showing that cell exposure to AgNPs induced significant cytotoxicity [138–141]. Conversely, Yen et al. determined a lower cytotoxicity of AgNPs than that of the AuNPs and attributed this difference to the surface charges between NPs, which can explain the discrepancy with other studies related to AgNPs cytotoxicity [135].

As for platinum, the cytotoxicity of 5–8 nm PtNPs capped with polyvinyl alcohol (PVA) has been addressed in human cells, where PtNPs were shown to enter the cells through diffusion, leading to an increase in DNA damage, proliferating cell nuclear antigen-mediated growth arrest and apoptosis [126]. Asharani et al. performed a comparison between toxicity of 3–10 nm Pt-, 5–35 nm Ag-, and 15–35 nm AuNPs capped with PVA in developing zebrafish embryos, concluding that AgNPs were the most toxic, followed by PtNPs, while AuNPs presented no indication of toxicity [142].

Even though we have focused our attention on the toxicity aspects of the different noble metal nanoparticles based mainly on size and metal, attention should also be brought upon other properties of the nanoconjugates, such as surface chemistry, shape, and administration pathways. In fact, surface chemistry (e.g., functionalization with biomolecules, stabilizers, etc.) constitutes another interface of interaction with the organism's proteins and cells, which in term may be associated with unspecific adsorption or specific recognition by the immune system, thus contributing to the overall effects of the use of the nanoparticles. The interaction with the immune system contributes not only for the specificity of the targeting (passive and/or active), but also towards the toxicological effect of nanoconjugates (see [122] and references therein).

## 5. Conclusions

Nanotechnology has provided for novel and powerful systems that may be used treatment and diagnostic of cancer. *In vivo* demonstrations of noble metal NPs as theranostic agents are now emerging and serve as important milestones towards clinical application. Nonetheless, the majority of products, reagents and drugs being used for the development of these nanoscale theranostic agents have still to be approved by the main supervising agencies, such as the FDA and EMA. Thus far, there are some questions whose answers still provide no clear understanding about the design and application of NPs, such as pharmacokinetics, biodistribution and side effects of the nanotherapy, and safety profile of NPs before and after conjugation and toxicity [10]. Are noble metal NPs cytotoxic or biocompatible? And how can the NPs be design to avoid these effects? These seem to question more difficult to answer than previously believed. Most therapeutic and imaging approaches based on noble metal NPs rely on AuNPs, mostly due to their higher level of nontoxicity. Nonetheless, a more comprehensive application of core/shell or alloy noble metal NPs, that may allow combination of the benefits of each noble metal into a single carrier, is still to be thoroughly addressed. Even though there is not any general mechanism for making NPs universally “nontoxic” to all living cells and all organisms, there are important findings that can be applied for increasing nanoparticle biocompatibility and reducing cytotoxic interactions *in vivo* and *in vitro*. In general, using the lowest NP dose to get the desired response for the shortest period of time seems to promote biocompatibility. The coating/capping of a nanoparticle is also of the utmost relevance, since a noncontinuous covering, the presence of cracks, roughness, or interruptions could lead to complement or antibody attachment, or dissolution of the coating by cell digestion, decreasing bioavailability at target cell [143]. It is essential to test nanoparticle/biological interactions experimentally and modify the NPs for best biocompatibility with the cell in order to eliminate damage to healthy tissue, guarding against alterations in genetic/molecular function while killing the abnormal cells. When interpreting NPs interactions with biological cells and organisms, it is important to remember that living systems may appear normal and be capable of growth and function, but they may be genetically altered in subtle ways following NP exposure, which can produce serious consequences at some time in the distant future, such as cancer itself.

Noble metal nanoparticles have shown to be powerful tools against cancer though still in need of further optimization and characterization for full understanding of their whole potential. It is now time to start translating these promising platforms to the clinical settings towards widespread effective therapy strategies in the fight against cancer.
